# Clinical Impact of *Staphylococcus aureus* Skin and Soft Tissue Infections

**DOI:** 10.3390/antibiotics12030557

**Published:** 2023-03-11

**Authors:** Matthew S. Linz, Arun Mattappallil, Diana Finkel, Dane Parker

**Affiliations:** 1 Department of Pathology, Immunology and Laboratory Medicine, Center for Immunity and Inflammation, Rutgers New Jersey Medical School, Newark, NJ 07103, USA; 2 Department of Pharmaceutical Services, University Hospital, Newark, NJ 07103, USA; 3 Division of Infectious Diseases, Department of Medicine, Rutgers New Jersey Medical School, Newark, NJ 07103, USA

**Keywords:** *Staphylococcus aureus*, skin-and-soft-tissue infections, antimicrobial resistance, bacterial pathogenesis, clinical presentation, treatment, antibiotics, epidemiology

## Abstract

The pathogenic bacterium *Staphylococcus aureus* is the most common pathogen isolated in skin-and-soft-tissue infections (SSTIs) in the United States. Most *S. aureus* SSTIs are caused by the epidemic clone USA300 in the USA. These infections can be serious; in 2019, SSTIs with *S. aureus* were associated with an all-cause, age-standardized mortality rate of 0.5 globally. Clinical presentations of *S. aureus* SSTIs vary from superficial infections with local symptoms to monomicrobial necrotizing fasciitis, which can cause systemic manifestations and may lead to serious complications or death. In order to cause skin infections, *S. aureus* employs a host of virulence factors including cytolytic proteins, superantigenic factors, cell wall-anchored proteins, and molecules used for immune evasion. The immune response to *S. aureus* SSTIs involves initial responders such as keratinocytes and neutrophils, which are supported by dendritic cells and T-lymphocytes later during infection. Treatment for *S. aureus* SSTIs is usually oral therapy, with parenteral therapy reserved for severe presentations; it ranges from cephalosporins and penicillin agents such as oxacillin, which is generally used for methicillin-sensitive *S. aureus* (MSSA), to vancomycin for methicillin-resistant *S. aureus* (MRSA). Treatment challenges include adverse effects, risk for *Clostridioides difficile* infection, and potential for antibiotic resistance.

## 1. Epidemiology

The bacterium *Staphylococcus aureus* is a commensal pathogen often present on the skin, nares, and mucous membranes of healthy individuals [1]. It represents a common global cause of human infection and easily acquires antimicrobial resistance through mutation or horizontal transfer of resistance genes from other bacteria [2]. Since the 1960s, methicillin-resistant *S. aureus* (MRSA) has been observed among hospitalized patients, and it has been spreading in the community since the 1990s [3]. MRSA has been associated with epidemic waves, with regional variants overtaking previously dominant strain types over time [4,5].

*S. aureus* is the most common pathogen isolated in cultures of skin-and-soft-tissue infections (SSTIs) in the United States [6]. Since 2000, the rise of community-acquired-MRSA (CA-MRSA) has been associated with a global increase in the incidence of *S. aureus* SSTIs [7,8,9,10,11]. With the increasing prevalence of CA-MRSA strains, there has also been an increase in virulent infections, including necrotizing fasciitis and necrotizing pneumonia [12]. However, MRSA SSTI hospitalization rates appear to have peaked around 2010, decreasing from 3.8 to 3.0 per 1000 hospitalizations in 2014 [13]. In 2016, the rate of MRSA-related SSTI hospitalization was 1.72 per 1000 hospitalizations, dropping significantly to 1.32 per 1000 by 2019 [14]. There are two likely factors contributing to falling SSTI hospitalization rates: a reduction in unnecessary antibiotic use, leading to reduced CA-MRSA transmission, and better SSTI management in the emergency department [14]. The overwhelming majority (97–99%) of community-onset SSTIs with *S. aureus* in the United States are caused by the epidemic clone USA300 [15,16,17]; USA300 also predominates in Canada [18], Columbia, Ecuador, Venezuela, and Samoa [19]. However, USA300 is not the dominant strain in Europe [20,21], Asia [22,23], or Australia [24]. Among healthy adults, recurrent SSTIs with *S. aureus* are common, affecting between 16–19% of patients; most adults have only one recurrence within 3 months of primary infection [25].

Risk factors for *S. aureus* SSTIs are well-characterized, particularly for methicillin-resistant strains. This includes a host of co-morbidities, such as cardiovascular disease, peripheral vascular disease, diabetes mellitus, renal disease, chronic wounds, immunosuppression, IV drug use, and presence of an abscess [26,27]. One large multicenter study developed a bedside risk score to discriminate between MRSA and non-MRSA SSTIs using the following factors: age (3 points if 30–39, 2 points up to 29 or 40–49, 1 point 50–59, 0 60+), Black race (1 point), no evidence of diabetes mellitus (1 point), no evidence of cancer or renal dysfunction (1 point), and prior history of cardiac dysrhythmia (1 point); a score higher than 5 exhibited superior accuracy in diagnosing MRSA compared to the healthcare-associated infection (HAI) classification. The HAI classification was defined as the presence of one or more of these four criteria: admission from chronic care, hospitalization in the prior 180 days or recent outpatient surgery, hemodialysis within 90 days or presence of end-stage renal disease, or chronic mechanical ventilation dependence [28]. The use of dialysis and central venous catheters are also associated with MRSA SSTIs [26]. Other *S. aureus* SSTI risk factors include prior colonization, infection, ICU admission, receipt of antimicrobial therapy, hospitalization in the prior year, and recent travel to Latin America, Africa, or Southeast Asia [26]. In the United States, children, people 65 years and older, and Black, Native American, and Multiracial patients have an elevated risk of CA-MRSA SSTIs [6,8,29], as do prisoners, athletes, and military personnel [29,30]. In the latter categories this can be attributed to close-quarter living. In the prison population, systematic review has also identified sharing soap, younger age (20–34 years old), low frequency of handwashing, poor hygiene, and being overweight as significant MRSA SSTI risk factors [31]. Pediatric patients are more likely to develop skin infections if they are colonized with MRSA, are Black, have had a household member with skin infection in the year prior, have chronic health issues, bite their nails [32], or have eczema [33]. For many of these risk factors, including race, further research into structural and social influences well as other findings such as obesity should be done.

## 2. Morbidity and Mortality

*S. aureus* infections can be highly dangerous, leading to death, regardless of their antimicrobial resistance patterns [34]. In 2019, *S. aureus*, along with *Escherichia coli*, *Streptococcus pneumoniae*, *Klebsiella pneumoniae*, and *Pseudomonas aeruginosa* accounted for 30.9% of 7.7 million infection-related deaths globally [35]. *S. aureus* alone was the leading bacterial cause of death in 135 countries and was associated with 1,105,000 deaths in 2019 [35]. MRSA was also the most common cause of death associated with antimicrobial-resistant infections in 27 countries in 2019; in 47 other countries, it was second to aminopenicillin-resistant *E. coli* [36]. In high-income countries, *S. aureus* and *E. coli* caused more than half of antimicrobial-resistant-infection-related fatalities in 2019, with MRSA responsible for more than 100,000 deaths and 3.5 million disability-adjusted life years [37]. Despite its staggering mortality and morbidity burden, *S. aureus* is listed as a “high priority” pathogen by the World Health Organization, not the highest designation, “critical priority” [38].

In 2019, *S. aureus* SSTIs were associated with 37,500 all-cause deaths and an all-cause, age-standardized mortality rate of 0.5 globally [35]. One study in the Netherlands found that *S. aureus* was the most commonly isolated pathogen in cellulitis and necrotizing fasciitis patients admitted to the intensive care unit [39]. Compared to sepsis and pneumonia patients, patients with *S. aureus* cellulitis tend to have low mortality rates [40]. There do appear to be some differences in outcomes between skin infections caused by CA-MRSA and other MRSA strains, with significantly lower rates of hospitalization for CA-MRSA patients [41], but no significant differences in need for surgical drainage [41,42], abscess size [42], treatment failure [41], or mortality [41]. One study in Australia found that patients with MSSA SSTIs were more likely to require IV antibiotics in the first 48 h of hospital admission and have a longer length of stay compared to CA-MRSA patients [42]. The existing data suggest that *S. aureus* SSTIs represent a serious health threat, regardless of their antimicrobial resistance patterns.

## 3. Economic Burden

In the United States, the burden of community-acquired *S. aureus* infections results mainly from hospitalization and mortality, with total costs of up to $13.8 billion annually [43]. Despite relatively low hospitalization rates, SSTIs account for a substantial portion of these costs [44]. In addition, these costs appear to be rising. In 2008, treatment for *S. aureus* SSTIs cost on average $8865 per patient [45]. In 2009, the average cost of *S. aureus* SSTI hospitalization was $11,622, marking a 30% increase in hospital expenditures within a year [46]; by 2011, the median charge for *S. aureus* SSTI hospitalization was $19,894 [47].

As CA-MRSA infections have become more common, with methicillin rates reaching up to 75% prevalence [48], there is evidence that they have contributed to rising healthcare costs for SSTIs [43,49]. However, the strength of the association between methicillin resistance and higher costs has been challenged in recent years [13]. From 2010 to 2014, costs for methicillin-sensitive *S. aureus*-related (MSSA-related) SSTI hospitalization ($11,098) were not statistically different from costs for MRSA-related SSTI hospitalization ($10,873) when adjusted for propensity score mortality outcomes [13]. Similarly, in a study adjusting for time-dependent bias among patients in a veteran database, statistical matching reduced inpatient excess variable and total cost estimates for MRSA healthcare-associated infections by 19.1% and 19.7%, respectively [50]. These analyses suggest that the failure to consider bias when estimating healthcare costs can lead to erroneous overestimation of the cost burden for *S. aureus* SSTIs. In a cohort study of women with postpartum *S. aureus* breast abscesses, the mean attributable cost varied from $2340 to $4012, with no significant difference between MRSA and MSSA infections [51]. A systematic review determined that while patients admitted to the hospital for MRSA pneumonia, bloodstream infections, or surgical site infections had increased median total hospital costs compared to MSSA infections [52], MRSA SSTIs had no significant difference in total hospital costs compared to MSSA infections [47,52]. Considered together, these studies indicate that MRSA infections may not increase healthcare costs as significantly as previously believed, but more longitudinal research is likely needed.

## 4. Clinical Presentations

### 4.1. Folliculitis

*S. aureus* is the most frequently isolated bacterium in folliculitis, which commonly affects the face, but can occur anywhere on the body with pilosebaceous units [53]. Folliculitis refers to inflammation of a hair follicle, which classically presents acutely with clusters of red papules < 5 mm in diameter [53]. *S. aureus* folliculitis may also manifest with superficial or deep infections. The superficial variant presents as small papules and pustules above an erythematous base, which typically heal without scarring. It may also appear as impetigo of Bockhart, with localized pustules (classically yellow pustules with hair penetrating them) that form clusters and then heal within one week, unlike streptococcal impetigo, which typically spreads [53,54]. Bockhart’s impetigo typically occurs after trauma or may be secondary to occlusion by emollient cream, topical steroids, or even sweating on a plastic waterbed [54]. The deep variant of folliculitis presents with plaques and nodules typically overlain by pustules. It causes tenderness and heals, but forms scars [53].

### 4.2. Boils/Furunculosis

*S. aureus* furunculosis represents a deep, necrotizing form of folliculitis that presents with tender, erythematous papules or nodules containing a central pustule [55]. Furuncles or boils are considered to be localized abscesses in the hypodermis [56]. Furuncles are more likely to occur in armpits and in the gluteal region, which are both rife with hair follicles [57]. *S. aureus* furunculosis has been associated with the Panton Valentine Leukocidin (PVL), a cytotoxic virulence factor, with positivity rates between 40 and 90 percent [55,58,59,60]. PVL-positive furuncles can be either simple or chronic and recurrent; PVL-positive furuncles are more common in young, healthy adults, while PVL-negative furuncles have been associated with diabetes, leukemia, and autoimmune disorders [59]. PVL-positive furuncles have also been associated with furunculosis outbreaks [58,61]. Chronic furunculosis is strongly associated with nasal colonization by *S. aureus* [58].

### 4.3. Carbuncles

Carbuncles represent the most severe variant of the hair follicle infection spectrum, usually forming from the coalescence of two or more furuncles. They are, like the other hair follicle infections, most often caused by *S. aureus* [62]. Extending more deeply into the hypodermis [56], they can form sinus tracts, and they may present in any part of the body with hair follicles, classically in the posterior cervical region, back, thighs [57], face, scalp, axillae, buttocks, and perineum [62]. Carbuncles are usually indurated and tender, also causing systemic manifestations like fever and chills. Patients with hyperhidrosis, dermatitis, or other immunocompromising conditions like diabetes, hypogammaglobulinemia, and chronic granulomatous disease have higher rates of carbuncles [62]. Carbuncles can be confused with *Candida* kerions or hidradenitis suppurativa [62]. Like furuncles, carbuncles are known to cause nosocomial outbreaks [63].

### 4.4. Impetigo

Impetigo is a skin infection impacting the keratin cells in the epidermis [56]. More common in warm and humid climates [64], it is usually caused by *S. aureus* or *Streptococcus pyogenes,* and typically affects children between the ages of two and five. Impetigo has bullous and non-bullous variants; the nonbullous syndrome is more common, presenting with a yellow crust on the face, arms, and legs that begins as a papule, becomes a vesicle, and then finally forms a central pustule. As the lesion heals, it typically forms a golden crust that persists for less than one week after initial symptoms appear. Nonbullous impetigo does not usually cause fevers, but it may be associated with pruritis and regional lymphadenopathy. The bullous syndrome typically presents in children younger than two with bullae on the arms, legs, and trunks, that rupture to form yellow scars. Impetigo may cause chronic infection, and it may result in other complications including acute poststreptococcal glomerulonephritis [56], a disease characterized by deposition of immune complexes in the kidneys. These complexes cause inflammation and symptoms including hematuria, edema, hypertension, and oliguria [65].

### 4.5. Erysipelas/Cellulitis

Erysipelas and cellulitis are overlapping syndromes, as erysipelas represents a type of cellulitis that is non-purulent and affects the epidermis [56]. Erysipelas extends only to the superficial dermis and associated lymphatic vessels [39]. They are thought to be more often caused by streptococcal infections than *S. aureus* [39,66,67,68], though one systematic review found that there was only a small difference between the frequency of positive cultures for both bacteria among cellulitis but not erysipelas patients [66]. Cellulitis, in contrast to erysipelas, extends to all layers of the skin; the two syndromes present similarly, with generalized symptoms including edema, erythema, and tenderness [39]. Cellulitis is sometimes confused for other skin maladies such as stasis dermatitis, stasis ulcers, gout, edema from congestive heart failure, or deep vein thrombosis [39].

### 4.6. Mastitis

Mastitis refers to inflammation of the breast tissue, which is frequent among breastfeeding women. It usually presents with localized tenderness as well as systemic fever and malaise [69]. *S. aureus* is one of the most common infectious causes [70] and the predominant agent in acute mastitis [71]. There are also subacute and granulomatous forms, for which *Staphylococcus epidermidis* and *Corynebacteria* species, respectively, have been identified as the most common etiologic agents [71]. Mastitis can result from mechanical irritation of the nipples by poor infant latching, infant conditions such as infection, or congenital anomalies such as cleft palate [69]. It may also result from milk stasis [71]. In one observational study of breastfeeding women, *S. aureus* was isolated in 50% of mastitis and 70% of breast abscess patients, respectively [72]. In this study, PVL-positive infections and MRSA strains were associated with higher hospitalization rates [72].

### 4.7. Folliculitis Decalvans

Folliculitis decalvans (FD) is a rare disease of the scalp associated with *S. aureus* infections. The exact role of *S. aureus* in pathogenesis is not completely understood, though an abnormal host response to the bacteria has been proposed as one mechanism [73]. FD presents with alopecia, tufting of hair, and pustules on the scalp [74]. Large studies on FD are limited. One comparative study comparing the microbiome of FD patients to healthy controls found that clearance of *S. aureus* with anti-staphylococcal treatment was associated with clinical improvement [74]. Another retrospective study found that *S. aureus* isolated from FD patients had increased resistance to macrolide and tetracycline antimicrobial agents [73].

### 4.8. Staphylococcal Scalded Skin Syndrome

Staphylococcal scalded skin syndrome (SSSS) is a syndrome that typically affects neonates and young children secondary to localized infections, though it may also occur in older patients with severe disease, such as pneumonia, septic arthritis, pyomyositis [75], immunosuppression, or renal disease [76]. It presents as a syndrome varying from localized, easily-ruptured bullae, to exfoliation throughout the body with systemic malaise, poor appetite, irritability, and generalized rashes [75]. Among the SSTIs caused by *S. aureus*, SSSS is unique in that it is mediated by exfoliative toxins A and B, which are the causative agents of symptoms [75,77]. Unless SSSS is quickly diagnosed, patients are at risk for other infections, in addition to sepsis and renal failure [78]. However, the prognosis for young children with SSSS is generally excellent. Most patients heal completely within 2 weeks; the extent of exfoliation does not impact outcomes [79].

### 4.9. Necrotizing Fasciitis

While necrotizing fasciitis, one of the most severe SSTIs, is classically associated with other pathogens such as group A streptococcus, *Clostridium perfringens*, or an assortment of mixed aerobic and anaerobic organisms, *S. aureus* can also cause monomicrobial necrotizing fasciitis [80,81,82]. The infection presents with local pain as well as systemic symptoms that may include fever or hypothermia, hypotension, and an altered mental status [83]. Risk factors include IV drug use, diabetes, cancer, and HIV/AIDS [80]. In a small study of necrotizing soft tissue infections with *S. aureus* and *S. pyogenes* in Iowa, necrotizing fasciitis was more common among white male patients [84]. After 2000, MRSA emerged as the major cause of necrotizing fasciitis associated with *S. aureus* infection, though there does not appear to be a significant difference in mortality rates for MRSA vs. MSSA necrotizing fasciitis [80,85]. Necrotizing fasciitis typically causes long term complications; these include a prolonged ICU stay, mechanical ventilation dependence and need for reconstructive surgery [80].

## 5. Pathogenesis

**Figure 1:**
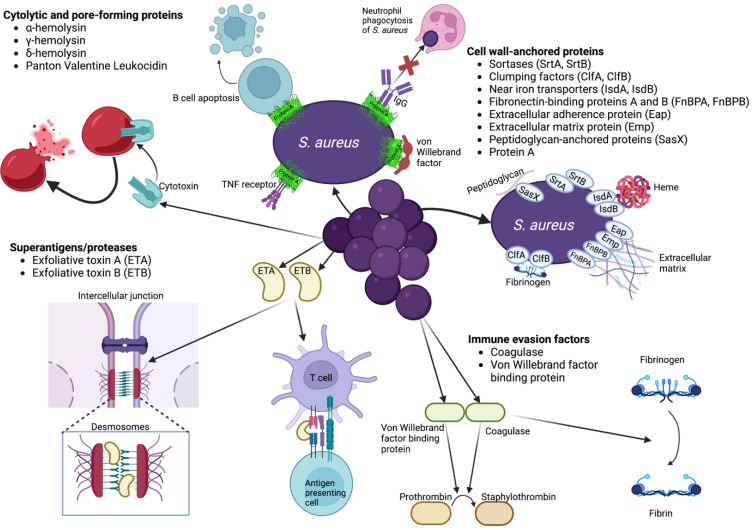
Major virulence factors involved in *S. aureus* SSTIs and some of their associated mechanisms.

The bacterium *S. aureus* produces a number of virulence factors thought to be important for skin infection (Figure 1), including cytolytic proteins (Table 1), superantigenic factors, molecules used for immune evasion (Table 2), and cell wall-anchored proteins (Table 3). In animal models, the staphylococcal cytotoxin ɑ-hemolysin, also known as Hla or ɑ-toxin, binds to the metalloprotease ADAM10 to initiate cell lysis [86]. Once activated, the ADAM10 metalloprotease cleaves E-cadherin, disrupting epithelial cell-cell adhesion and cell migration [87]. In mastitis and skin infection models in mice, *Δhla* mutants exhibit reduced virulence with less severe histopathologic changes and smaller lesions with minimal dermonecrosis, respectively [88,89]. However, the role of Hla in human SSTI virulence has not been well characterized [90]. In an infection model using immortal human HaCaT keratinocytes, a fraction of USA300 mutants that lacked the *hla* or *agr* genes had longer intracellular survival than WT [91]. In another *S. aureus* cutaneous infection model using human skin explants, the promoter for the *hla* gene was only activated in sweat glands and ducts at 2 h post-infection, not on the skin’s surface [92]. In the same model, genes for surface proteins like *sasC*, *sasD*, *sasG*, *sasB*, *clfB* (clumping factor B), and *spa* (surface protein A) are expressed early on, while genes encoding proteins for regulation of transcription like *agrA*, *mgrA*, and *sarZ* are upregulated after 24 h. Secreted factors such as alpha-hemolysin, aureolysin, and the type 7 secretion system extracellular protein C, remain constitutively expressed throughout infection [92]. During colonization and early infection, ferric hydroxamate uptake D2 lipoprotein (iron uptake) and leukotoxin ED (neutrophil killing) are upregulated, while adenosine synthase A is downregulated [92]. Other virulence factors upregulated later on during infection include V8 protease and staphopain B [92].

*S. aureus* uses bicomponent pore-forming toxins such as γ-hemolysin, δ-hemolysin, PVL, and LukED, which can lyse polymorphonuclear neutrophils (PMNs) and other leukocytes [93]. All three, as well as α-toxin, are expressed by epidemic MRSA strains on human skin mimic models [94]. The γ-hemolysin has three parts that form two leukocidin proteins, HlgAB and HlgCB, which both bind to human receptors, but their specific role in virulence is not yet clear [95]. HlgCB mediates increased bacterial load in mouse skin infections [96], but a better understanding of HlgAB has been impeded by limitations of existing in vivo mouse models [95]. The staphylococcal δ-hemolysin also has non-specific pore-forming activity [97]. In addition, δ-hemolysin induces degranulation of mast cells in mice, which likely plays a role in the pathogenesis of atopic dermatitis [98]. In a humanized mouse model of pneumonia, infection was more severe with a PVL-positive USA300 WT strain than with PVL mutants [99]. While a systematic review found that PVL genes are strongly associated with *S. aureus* SSTIs [100], the specific role of PVL in USA300 skin infections remains unclear, with inconsistent findings across studies in mice, rabbits, monkeys, and human keratinocytes [101,102,103,104,105]. However, some recent data using humanized mice is promising. Using non-obese diabetic (NOD)/severe combined immune deficiency (SCID)/Il2 rγ^null^ (NSG) mice that were engrafted with human CD34^+^ umbilical cord blood cells, Tseng et al. showed that PVL-positive *S. aureus* induced larger skin lesions than PVL mutant strains despite similar CFU burdens. When the NSG mice were injected with human neutrophils, PVL-positive *S. aureus* similarly induced larger lesions than PVL mutant strains, again with similar CFU counts [106]. In addition to pore-forming toxins, *S. aureus* expresses several superantigen proteins, which activate T cells by crosslinking the T cell receptor with MHC class II molecules to disrupt the physiological immune response [107]. In SSSS, the exfoliative superantigen toxins ETA and ETB induce polyclonal expansion of T cells [108]. In the presence of Ca^2+^, *S. aureus* ETA can specifically bind to desmoglein 1 [109], but not other desmogleins, thus specifically targeting desmosome cell-cell adhesion in the epidermis, which, when disrupted, creates blisters of the superficial skin [110]. As a serine protease, ETA has great specificity and only disrupts cell adhesion in the superficial epidermis [110]. The same is true for ETB, which specifically cleaves desmoglein 1 but not desmoglein 3 in the superficial epidermis [111].

Cell wall-anchored proteins are thought to play a critical role in *S. aureus* skin infections, though further research into individual factors is needed. The cell wall-anchored proteins with known functions are classified by their primary motifs, including clumping factors that can bind fibrinogen and desquamated epithelial cells (ClfA, ClfB, SdrC, SdrD); near iron transporters that bind and transport heme (IsdA, IsdB); and protein A that can bind many different molecules and evade the immune response [114,141]. In mouse models of skin abscess and dermonecrosis, knockout strains for the cell wall-anchored proteins sortase A and B (which covalently link surface proteins to the cell wall) display less swelling, and have lower skin CFU counts and necrosis, respectively, compared to the WT strains [115]. ΔClfA bacteria yield reduced CFUs in mouse skin abscesses, and a strain engineered to have ClfA unable to bind to fibrinogen exhibited an even steeper decrease in bacterial burden [115]. Monitoring the *S. aureus* LAC strain transcriptome in a rabbit skin infection model, Malachowa et al. found that near iron transporter IsdB was upregulated about 125-fold at 24 h post-infection, with upregulation of other cell wall-anchored proteins like SdrE (a fibronectin-binding protein, 12-fold), efb (a fibrinogen-binding protein precursor, 5-fold), and SAUSA300_1052 (a fibrinogen-binding protein, 6-fold) [105]. Antibodies to IsdA and IsdB are protective against staphylococcal abscess formation in mice, likely by disrupting *S. aureus’s* ability to scavenge heme [117]. In rhesus macaques, a vaccine using IsdB is highly immunogenic, increasing antibody titers more than 5-fold [118]; however, an IsdB candidate vaccine from Merck showed futility in phase 2 and 3 trials [119]. An intravenous mouse infection model determined roles for ClfA and ClfB in the early dissemination of *S. aureus*, as well as roles for IsdA, IsdB, SdrD, and protein A in abscess formation [116]. While mutants for each of these genes had lower bacterial loads, ΔClfA and ΔClfB mutants still had intact abscess formation, likely reflecting a role in fibrinogen binding and pathogen survival in early infection [116].

Other cell wall-anchored proteins critical for *S. aureus* SSTI pathogenesis include fibronectin binding proteins and other proteins that bind to molecules in the extracellular matrix as well as SasX, which is likely covalently anchored to peptidoglycans via an LPXTG motif. Fibronectin binding proteins are thought to permit *S. aureus* persistent and chronic infections by mediating bacterial internalization into keratinocytes via the fibronectin-binding integrin α5β1 [123], although there are alternative internalization mechanisms independent of fibronectin binding proteins [124]. *S. aureus* also upregulates fibronectin binding proteins that induce Hla-ADAM10-mediated up-regulation of β1 integrins to increase internalization within mast cells in mice in order to evade extracellular antimicrobial activity [125]. One recent study determined that fibronectin binding protein B (FnBPB) also facilitates adherence of *S. aureus* to loricrin, the protein that serves as the initial adhesion site for bacteria during skin colonization in humans [120]. Similarly, mutants of *S. aureus* lacking FnBPB demonstrated reduced adherence to corneocyte skin cells from human patients [121]. When *S. aureus* made initial contact with ex vivo human skin explants, the expression of fibronectin binding protein A was strongly upregulated [122]. Also important for *S. aureus* skin infections is the extracellular adherence protein (Eap), used for adhering to and invading host cells by interacting with the extracellular matrix [124,126]. When HaCaT cells or other human keratinocytes were incubated with Eap and then exposed to *S. aureus*, the number of infected cells and bacterial load per infected cell significantly increased, as confirmed by flow cytometry [124]. Similarly, *S. aureus* employs the extracellular matrix protein (Emp) to bind human skin sample extracellular matrix in vitro [128]. All three—fibronectin binding proteins, Eap, and Emp—play a role in *S. aureus* abscess formation [90,116], and Eap and Emp also contribute to biofilm formation under low-iron conditions [127]. In recent years, the surface protein SasX, which is encoded by mobile genetic elements, has also been identified as a key virulence factor for MRSA skin infections [129,130]. In mice skin infections with *S. aureus*, immunization with recombinant SasX or rabbit polyclonal anti-SasX IgG reduced lesion size and decreased MRSA colonization, also increasing neutrophil killing [131].

The role of the virulence factors Eap and Emp in biofilm formation is clinically relevant, as biofilms can protect bacteria against the immune system as well as antibiotics. These biofilms can occur not only with foreign body infections, but also with chronic wounds or recurrent infections of heart tissue or cartilage [142]. Biofilms formed by *S aureus* are surrounded by a slime glycocalyx layer made up largely of polysaccharide intercellular antigen (PIA), which is produced by the *ica* intercellular adhesion locus [143]. However, certain strains form biofilms independent of the *ica* locus [144]; other factors implicated in *S. aureus* biofilms include protein A [145], fibronectin binding proteins [146], autolysin [146], extracellular DNA [146,147], and biofilm matrix proteins like Bap [148]. In other bacteria that can form biofilms on the skin such as *S. epidermidis*, biofilms are also mediated by PIA production. However, certain strains also have PIA-independent biofilm pathways, such as the icaC::IS256 mutant, which formed biofilms using accumulation-associated protein Aap and downregulated the homologous Bap protein [149,150]. In *Staphylococcus lugdunensis*, one study of clinical isolates found that only one of 11 strains was icaA-positive and formed biofilms [151]. Unlike those formed by staphylococcal species, *S. pyogenes* biofilms do not appear to require polysaccharides [152], and they exhibit strain-specific variation in required components for biofilm development, including pili [153], M protein, and streptococcal protective antigen [154]. The characterization of the many virulence factors involved in *S. aureus* biofilm formation and mechanistic studies for agents known to disrupt biofilms represent a source of active investigation.

In addition to toxins and cell wall-anchored proteins, *S. aureus* also uses immune evasion factors like coagulase (Coa) and von Willebrand factor binding protein (vWbp) during SSTI. Both molecules can activate prothrombin, and Coa is also able to convert fibrinogen to fibrin by proteolysis. Both proteins are known to contribute to abscess formation, likely by helping to form a fibrous capsule at the periphery of the abscess [90]. In one study, mutant strains without coagulase activity did not exhibit reduced virulence when used for subcutaneous and intramammary infections in mice [112]. However, more recent studies in rabbits found that mutant *S. aureus* strains with isogenic deletion of Coa or vWbp caused significantly smaller skin abscesses than WT strains [113]. A similar decrease in abscess size was seen in strains deficient in both Coa and vWbp, though there was no clear mechanism underlying these observations [113].

The cell wall-anchored protein A has five triple helix domains at its N-terminus, used for binding IgG, TNF receptor 1, and von Willebrand factor [114]. It is critical for evading immune recognition and response, binding IgG to escape phagocytosis by neutrophils [132,139,140] and acting as a superantigen for B cells to induce apoptosis [133]. In vitro experiments using human keratinocytes and immortal HaCaT keratinocytes showed that exposure to purified protein A increased expression of proinflammatory genes *COX2* and *IL8*; however, when keratinocytes were matched to patients, there was no correlation between in vitro findings and skin symptom severity [134]. These results are consistent with other studies that have failed to associate the presence of protein A with skin inflammation in atopic dermatitis patients [135]. However, more recent studies suggest a mechanism for protein A in atopic dermatitis virulence. In 2017, Jun et al. found that in atopic dermatitis patients, protein A penetrates the skin barrier to form membrane vesicles that increase expression of pro-inflammatory cytokines IL-1β, IL-8, MIP-1ɑ, and IL-6 in keratinocytes in vitro [136]. Another recent study showed that in mice infected with *S. aureus* lacking protein A, skin lesions were larger in the mutant compared to WT [137]. Mice infected with the mutant protein A strain also exhibited higher ratios of neutrophil necrosis to apoptosis and overall dermonecrosis [137]. For human keratinocytes, while Hla is needed for infection as it induces keratinocyte necrosis through a Ca^2+^-dependent calpain protease pathway, protein A expression is dispensable; SpA^-^ (protein A) mutants invade with the same ability as a WT USA300 strain [138]. The full role of protein A in skin infection has likely not been fully characterized.

## 6. Immune Response

During early infection, neutrophils predominate in the *S. aureus* SSTI immune response, giving way to other cell populations including antigen-presenting macrophages and dendritic cells, non-natural killer innate lymphoid cells, CD4^+^ Th17 cells, CD8^+^ T cells, and γδ T cells in later infection [155]. Early infection cytokines and chemokines consist of proinflammatory IL-6, CXCL1, CCL2, CCL3, G-CSF, GM-CSF, and IL-1β; later on, IL-17A, IL-17F, and CCL4 levels increase [155]. Many other cytokines also contribute to the immune response, such as IL-33, which activates NADPH oxidase to produce reactive oxygen species, inducing the production of neutrophil extracellular traps and reducing the bacterial burden [156]. In response to mechanical injury to skin, basophils produce IL-4, which likely assists *S. aureus* in evading the cutaneous immune response by suppressing defense mechanisms in keratinocytes and TCRγδ^+^ cells mediated by IL-17 [157]. Acute and chronic *S. aureus* infections appear to instigate similar immune responses, though the overall host response generally diminishes with time. In an acute SSTI mouse model, chemokines and factors associated with neutrophils were most upregulated, such as CXCL2, CXCL5, CXCL9, MMP9, and BAFF. In a related chronic biofilm mesh infection mouse model, CXCL2 and CXCL9 persisted, but the other cytokines were replaced by IL-1A and IL-17A, suggesting an early Th1 and Th17 response [158].

The innate immune response to *S. aureus* SSTIs is complex and incompletely understood. The skin immune response begins as keratinocytes react to trauma and bacterial exposure by producing antimicrobial peptides [159,160,161]; however, some bacteria are tolerated and permitted to colonize the skin [160]. Among other pattern recognition receptors (PRRs) such as TLR2 that mediate immune recognition of pathogens such as *S. aureus* [162], the innate immune sensor NOD2 displays specific recognition of *S. aureus* as a distinct pathogen from commensal microbes [163]. In a cutaneous infection model, *Nod2* knockout mice developed ulcers with 5- to 10-fold higher bacterial CFUs and increased NF-ΚB activity yet showed no difference for neutrophil count on histology compared to WT [163]. However, cutaneous infection with the commensal *S. epidermidis* failed to upregulate NF-ΚB activity [163]. Further, it seems that keratinocytes internalizing viable *S. aureus* and its dsRNA—not dead bacteria or *S. epidermidis*—stimulate the start of the innate pro-inflammatory immune response to *S. aureus* SSTIs [160]. In humans, Langerhans cells can also discriminate *S. aureus* from other staphylococcal bacteria with their unique receptor langerin, which can recognize *S. aureus* cell wall teichoic acid [164].

In response to *S. aureus* SSTI, activation of PRRs and the inflammasome (a protein complex that responds to microbial and molecular danger signals by producing pro-inflammatory cytokines) [165], IL-1β is produced, predominantly by neutrophils rather than dendritic cells or monocytes [166]. This IL-1β is sufficient to generate neutrophil abscesses and promote clearance of *S. aureus* skin infection in mice [166]. From there, resident skin cells, not bone marrow-derived cells, use MyD88-and IL-1R-dependent signaling pathways to promote further neutrophil recruitment [167]. However, while monocytes have a limited role in the early SSTI immune response, they are critical for augmenting the neutrophil response, responding in a MyD88-dependent fashion to help clear infection [168]. In mice, the scavenger receptor CD36, which is present on macrophages, is critical for controlling dermonecrosis in *S. aureus* SSTIs, also limiting the expression of pro-inflammatory IL-1β and myeloperoxidase from neutrophils, which contribute to skin injury [169]. However, while neutrophils are ultimately phagocytosed by resident dendritic cells, they must first migrate to skin draining lymph nodes, guided by upregulation of CCR7 on their surfaces [170].

Adaptive immunity against *S. aureus* SSTIs is not understood as well. *S. aureus* infections generate B and T cell responses in the host, yet protective immunity is not typically observed, nor is there an effective *S. aureus* vaccine. In BALB/c mice, primary *S. aureus* SSTI protected against secondary infection mediated by antibody-dependent and antibody-independent mechanisms, the latter likely mediated by T cells, as suggested by smaller lesions with T cell transfer from immune to primarily infected mice [171]. The same robust protective immunity was not observed in C57BL/6 mice; in humans, a similarly broad spectrum of immune responses to *S. aureus* infections with some genetic basis might account for the failure to create a universal *S. aureus* vaccine [171,172]. In another mouse model using C57BL/6 mice, mice reinfected with a severe ear SSTI after an initial epicutaneous *S. aureus* infection displayed a lower bacterial burden and progression of infection. This enhanced immune response depended on IgE effector mechanisms including mast cells; IgE-and FcεRIα-deficient mice lacked the protective immunity otherwise seen in this model, even as they had otherwise intact humoral responses besides IgE for the first group of knockout mice [173].

Adaptive T cell immunity against *S. aureus* SSTIs is dependent on clonal expansion of Vγ6^+^Vδ4^+^ T cells, which produce the redundant IL-17 cytokines, IL-17A and IL-17F, as well as IL-22 [174]. Of note, IL-17 from epidermal Vγ5^+^ γδ T cells recruits neutrophils to form abscesses, and it therefore also plays a role in the innate immune response [175]. Clonally expanded γδ T cells are induced by a pathway involving TLR2 and MyD88, producing TNF and IFN-γ to help protect against reinfection of the skin with *S. aureus* [176]. After skin injury, dendritic epidermal T cells, a prototypic form of intraepithelial lymphocytes, produce IL-17A to promote wound healing via β-defensin 3, RegIIIγ, and other antimicrobial peptides [177].

Adaptive immunity against *S. aureus* likely plays a role in atopic dermatitis as well. One study found that half of atopic dermatitis patients had IgE antibodies to *S. aureus* cell wall proteins, though elevated serum levels did not clearly correlate with skin symptoms, only regional lymphadenopathy [178]. In a group of atopic dermatitis patients with known sensitization of IgE against fibronectin-binding protein 1, immunodominant peptides from that virulence factor induced T helper cells to produce pro-inflammatory cytokines like IL-4 and IL-13, suggesting a type 2 immune response that may contribute to persistent, allergic inflammation [179].

## 7. Treatment

### 7.1. Selection of Antimicrobial Therapeutic Agents

Antimicrobial therapy against *S. aureus* SSTIs is delineated within several clinical treatment guidelines [180,181,182,183,184]. These resources include management considerations for non-purulent and purulent presentations of SSTIs. In addition, the United States Food and Drug Administration (FDA) provides guidance in the clinical development of systemic drugs for SSTIs, including efficacy considerations [185]. Studies to date have not established a superior antimicrobial agent in SSTIs [186,187,188]. The selection of an antimicrobial drug is typically dependent on disease presentation, local antimicrobial susceptibility patterns, and patient-centric factors (e.g., tolerance, adherence, and acquisition) [187].

### 7.2. Antimicrobial Therapeutic Agents Options

#### Overview

Antimicrobial drugs typically utilized in clinical management of SSTIs are designated within the following classes: first generation cephalosporins, penicillinase-resistant penicillins, novel cephalosporins, tetracyclines, glycopeptides, lipopeptides, lincosamides, oxazolidinones, and dihydrofolate reductase inhibitors [189]. In the management of MSSA, preferential options include first generation cephalosporins (e.g., cefazolin) and penicillinase-resistant penicillins (e.g., nafcillin and oxacillin) [187]. Studies indicate superiority of these agents in the management of invasive MSSA infectious presentations (not related to SSTIs) [190,191]. SSTIs due to MRSA requiring hospitalization are often managed with vancomycin, with alternative antimicrobial drugs being initiated when vancomycin use is not possible (i.e., safety, tolerance, and resistance) [180,181,187].

### 7.3. Commonly Utilized Therapeutic Agents

#### 7.3.1. Sulfamethoxazole-Trimethoprim (TMP/SMX)

The 2014 update of the Infectious Diseases Society of America (IDSA) SSTI guidelines recommend the use of TMP/SMX for purulent uncomplicated SSTIs [181]. Systematic reviews of investigations utilizing TMP/SMX support the efficacy of TMP/SMX with the presence of purulence, typically indicative of MRSA [192]. In most non-purulent SSTIs, several trials indicate a nonsignificant difference between TMP/SMX versus β-lactam agents (e.g., cephalexin), with the likely implication that treatment does not improve anti-MRSA antimicrobial activity [193,194,195]. Global surveillance continues to indicate >90% susceptibility of *S. aureus* to TMP/SMX, providing high confidence for continued empiric use in SSTIs [196]. Limitations precluding the use of TMP/SMX include: commonly reported adverse reactions (e.g., gastrointestinal intolerance, rashes, and hyperkalemia) and drug allergies (e.g., sulfa moiety) [197].

#### 7.3.2. Clindamycin

While trial data established the efficacy of clindamycin in SSTIs, surveillance results continue to indicate increased nonsusceptibility of MRSA isolates to clindamycin [194,196]. This pattern generally prohibits empiric utilization of clindamycin in purulent SSTI management. Additionally, inducible clindamycin resistance (ICR) mediated by ribosomal methylase behooves local laboratories to test for ICR on clinical *S. aureus*, as treatment failure from ICR isolates is reported [198,199,200,201]. However, in culture-directed therapeutic management, clindamycin continues to be an effective option [181]. Commonly reported adverse reactions primarily include gastrointestinal intolerance [197].

#### 7.3.3. Doxycycline/Minocycline

Second generation tetracycline class agents, including doxycycline and minocycline, are recommended for use in purulent uncomplicated SSTIs with a trial resulting in similar outcomes in patients when compared to TMP/SMX [181,202]. Surveillance results indicate a growing resistance to tetracycline, with 83% retained susceptibility against MRSA (94% with MSSA), while doxycycline is reported to have 90% susceptibility against MRSA (99% with MSSA) [196]. Commonly reported adverse reactions primarily include gastrointestinal intolerance and photosensitivity [197].

#### 7.3.4. Linezolid/Tedizolid

Evaluations of oxazolidinone class agents including linezolid and tedizolid in uncomplicated and complicated SSTIs continues to indicate favorable treatment outcomes along with potent retained susceptibility against *S. aureus* isolates [196,203,204,205]. Few pooled analyses suggest linezolid and tedizolid have significantly better outcomes versus vancomycin, however bias implications are noted in these primarily manufacturer-sponsored trials and inherent limitations of pooled analyses [206,207]. Limitations for the routine use of oxazolidinone class agents include: medication acquisition (i.e., cost), commonly reported adverse reactions (e.g., myopathy/rhabdomyolysis), and antimicrobial stewardship (i.e., use restriction to minimize emergence of resistance) [197].

#### 7.3.5. Vancomycin

Vancomycin continues to remain the general standard of care in the management of complicated SSTIs [180,181,182,183,184]. While susceptibility to vancomycin generally remains ~99% for *S. aureus*, some reports have indicated an increasing number of MRSA isolates with high glycopeptide MICs within the susceptible range, often designated at “Vancomycin MIC Creep” [196,208]. This phenomenon may also be accompanied by suboptimal clinical outcomes, including treatment failure [209,210,211]. However, analyses suggest the incidence of the “Vancomycin MIC Creep” may be near nil, not warranting concern regarding the efficacy of vancomycin in managing SSTIs [196,212]. Intravenous infusion reactions to vancomycin and therapeutic drug monitoring required to minimize the risk of drug-induced nephrotoxicity are the primary deterrents for convenient use [197,213].

#### 7.3.6. Daptomycin

Daptomycin is typically utilized as an alternative to vancomycin in the management of complicated SSTIs [180,181,182,183,184]. Analyses of treatment trials indicate similar treatment outcomes for SSTIs when compared to standard of care (e.g., vancomycin) [214,215]. Limitations precluding routine use of daptomycin include: medication acquisition, commonly reported adverse reactions (e.g., anemia, thrombocytopenia, and neutropenia with prolonged use), drug interactions (e.g., serotonergic potentiation), and antimicrobial stewardship [197].

#### 7.3.7. First Generation Cephalosporins/Penicillinase-Resistant Penicillins

First generation cephalosporins (e.g., cefazolin and cephalexin) and penicillinase-resistant penicillins (e.g., nafcillin, oxacillin, and dicloxacillin) are recommended for utilization in culture-directed SSTIs caused by MSSA [180,181,182,183,184]. Investigations indicate superiority of these agents in the management of invasive MSSA infectious presentations (not related to SSTIs) [190,191]. When compared to nafcillin for treatment of MSSA infections, cefazolin is reported to be better tolerated, with fewer adverse and cessation of therapy events [216]. However, there is an ongoing clinical impasse on the utility of cefazolin in the presence of high inoculum infection (e.g., highly invasive presentation) causing inducible resistance with the potential of treatment failure, while several reports indicate no difference in treatment outcomes [216,217,218].

### 7.4. Infrequently Utilized Therapeutic Agents

#### 7.4.1. Ceftaroline

Ceftaroline is an oxyimino cephalosporin with activity against MRSA utilized in the management of complicated SSTIs [219]. Primary analysis of Phase 3 studies utilizing ceftaroline when compared to standard treatments (including vancomycin or linezolid) indicate comparable outcomes for MRSA-related SSTIs [219]. Post-marketing analyses indicate similar treatment benefits with ceftaroline [220]. The most common side effects include rashes, pruritus, pyrexia, gastrointestinal intolerance, and infusion site reactions [197]. Limitations for routine use of ceftaroline include medication acquisition and antimicrobial stewardship. However, ceftaroline (along with other β-lactams) may have a niche role as an adjunctive treatment for persistent MRSA bacteremia [221,222,223,224]. Future clinical trial platforms may provide definitive answers on the utility of adjunctive β-lactam therapy for persistent MRSA bacteremia [225].

#### 7.4.2. Dalbavancin/Oritavancin

Long-acting (average terminal serum half-life ~250 h) intravenous lipoglycopeptides including dalbavancin and oritavancin offer convenience in single dose administration against *S. aureus* SSTIs [226,227]. Review of SSTI treatment trials indicate both lipoglycopeptides offer comparable safety and efficacy outcomes to standard comparators [228]. The convenience offered by lipoglycopeptides promotes utilization in treatment of SSTIs when standard options are not feasible (e.g., intolerance and inconvenience). Post-marketing observational use of lipoglycopeptides has been reported for invasive *S. aureus* infections, including infective endocarditis, osteomyelitis, and prosthetic joint infections [229]. Prospective investigations are required to assess the value (e.g., optimal dosing, safety, and outcomes) of these agents for invasive *S. aureus* infection [230].

#### 7.4.3. Tigecycline/Omadacycline

Third generation tetracyclines including tigecycline and omadacycline are generally considered an alternative treatment option for SSTIs, given the availability of several standard comparator options [231]. Both tigecycline and omadacycline have antimicrobial activity against some Gram-negative pathogens, in addition to *S. aureus*, which may be of benefit in patients with mixed-pathogen SSTIs [231]. Limitations discouraging routine use of third generation tetracyclines include gastrointestinal intolerance, medication acquisition, and antimicrobial stewardship [197,231].

#### 7.4.4. Delafloxacin

While fluoroquinolone class agents including ciprofloxacin, levofloxacin, and moxifloxacin are not routinely recommended in the management of *S. aureus* SSTI, delafloxacin recently demonstrated efficacy in SSTI treatment trials [180,181,182,183,184,232,233]. Delafloxacin offers coverage against MRSA, while also maintaining broad Gram-negative coverage typically offered by fluoroquinolones (e.g., *Pseudomonas aeruginosa*, *Enterobacterales*) [234]. Limitations preventing routine delafloxacin use include: medication acquisition, serious class-specific reported adverse reactions (e.g., aortic aneurysm risk, tendinopathy, peripheral neuropathy, and mental health adverse events), and antimicrobial stewardship [235]. Nonetheless, delafloxacin’s role may be limited to select presentations with limited standard treatment options.

#### 7.4.5. Rifampin

Rifamycin class agents including rifampin are not advocated as monotherapy in the management of infections caused by *S. aureus*, with more recent investigations finding no benefit to the adjunctive addition of rifampin to invasive *S. aureus* [180,181,233]. However, the protein synthesis inhibition exerted by rifampin may offer it a limited role in managing toxin-producing invasive MRSA infections [180].

### 7.5. Route of Treatment Administration and Duration

Most non-severe SSTIs can be managed with the utilization of oral therapy, where as severe SSTIs will initially necessitate the use of parenteral therapy. Conversion to oral therapy from parenteral therapy after initial positive responses to treatment is advocated to reduce adverse events and the duration of inpatient hospitalization [236,237]. In general, treatment duration should be based on individual response to treatment [181]. Several clinical trials have indicated non-inferior efficacy with shorter durations of treatments (e.g., 5–7 days) versus longer durations (e.g., 10–14 days) of uncomplicated, non-severe SSTIs [205,238,239].

### 7.6. Phage Therapy

Given the high capacity of *S. aureus* to develop a resistance to widely used antimicrobials, agents derived from bacteriophages have been proposed as an alternative or adjunct treatment approach [240]. In general, these phage therapies are available from academic or commercial sources. For SSTIs, topical phage administration is an option [240,241]. In mice, the JD007 phage-derived cell wall hydrolase lysin was able to eliminate intracellular MRSA in keratinocytes, limit MRSA proliferation in the skin, and facilitate wound healing of cutaneous abscesses when fused to a cell-penetrating peptide [242]. Rats with thigh soft-tissue MRSA infections treated with a phage cocktail entrapped in a nanostructured lipid-based carrier resolved infection within 7 days compared to 20 days for untreated animals [243]. In a case report of three Georgian lumberjacks who developed *S. aureus* radiation wound infections, PhagoBioDerm, a wound-healing preparation with polymers impregnated with ciprofloxacin and a mixture of bacteriophages, was successfully used to achieve clinical improvement and elimination of *S. aureus* within 7 days [244]. While preliminary data is promising [245], a panel of infectious diseases and bacteriophage experts in 2022 recommended that phage therapy should be limited to the treatment of infections refractory to antibiotic therapy, including MDR or hardware-source infections [240].

### 7.7. Treatment Challenges and Considerations

Systemic antimicrobial therapy can be associated with adverse effects such as allergic and hypersensitivity reactions, hematologic abnormalities such as thrombocytopenia, gastrointestinal effects and elevations of liver enzymes, and clostridium difficile infection. Increased use of systemic antibiotics can also potentially lead to antibiotic resistance [246]. However, though a few studies have suggested that incision and drainage of soft tissue infections alone may be adequate, several large studies identified that systemic antibiotic treatment in conjunction with post incision and drainage improved clinical outcomes compared to placebo for *S. aureus* infections and decreased reoccurrences [247,248].

## 8. Conclusions

*S. aureus* remains the most common pathogen causing SSTIs. While the hospitalization rate from *S. aureus* SSTIs appears to be decreasing in the United States, these infections still represent a serious healthcare challenge. *S. aureus* causes significant morbidity and mortality, representing the leading bacterial cause of death in 135 countries and contributing to more than a million deaths in 2019. Most presentations of *S. aureus* SSTIs can be managed with oral therapy due to their superficial nature, however these can easily develop into more invasive infection where parenteral therapy is needed. Given the rise of antimicrobial-resistant strains, experimental approaches are emerging for infections refractory to antibiotics, such as bacteriophage therapy. MRSA alone was responsible for more than 100,000 deaths worldwide in 2019, and *S. aureus* SSTIs had an all-cause, age-standardized mortality rate of 0.5. Areas needing further investigation are understanding the host factors and pathways that are important in responding to *S. aureus* infection. By understanding the host processes involved, immunomodulation strategies can be developed, which would be effective even for the most antimicrobial recalcitrant strains. Much less is also known about the genetic factors that can influence susceptibility to infection. Our understanding of virulence factors that *S. aureus* employs in skin infection, as well as the immune response to these bacteria, is still growing. While many cytolytic factors and adhesion factors have been characterized, a further appreciation of what bacterial factors are important is required. Therapeutics that can target some of these bacterial processes, such as biofilm formation, would represent another novel class of therapeutics. Some of these gaps in knowledge have potentially hindered vaccine efforts, such as the adaptive-immunity-hindering properties of protein A. *S. aureus* remains a formidable clinical foe that continues to play a large clinical and economic role in SSTIs.

## Figures and Tables

**Table 1:** Key cytolytic virulence factors understood to be important for *S. aureus* skin-and-soft-tissue infection.

Virulence Factor	Function	Models	References
Cytolytic and pore-forming proteins			
ɑ-hemolysin (Hla, ɑ-toxin)	General cell lysis	Mouse skin, mastitis	[86,87,88,89,90,91,92]
		Human HaCaT cells, skin explants	
γ-hemolysin (HlgAB, HlgCB)	Lysis of neutrophils, other leukocytes	Mouse skin	[93,94,95,96]
		Human skin mimic models	
δ-hemolysin (δ-toxin)	General cell lysis	Mouse skin	[97,98]
	Mast cell degranulation		
Panton Valentine Leukocidin (PVL)	Lysis of neutrophils, other leukocytes	Mouse skin	[99,100,101,102,103,104,105,106]
		Rabbit skin	
		Monkey skin	
		Humanized mice	
		Human keratinocytes	

**Table 2:** Key superantigen/protease and immune evasion virulence factors involved in *S. aureus* skin-and-soft-tissue infection.

Virulence Factor	Function	Models	References
Superantigens/proteases			
Exfoliative toxins (ETA, ETB)	Crosslink T cell receptor with MHC class II molecules Proteolysis of epidermal desmoglein 1	Mouse lymphocytes, skin	[107,108,109,110,111]
		Rabbit intravenous	
		Human lymphocytes, skin cryosections	
Immune evasion factors			
Coagulase (Coa)	Activation of prothrombin	Mouse skin, mastitis Rabbit skin	[90,112,113]
	Proteolytic conversion of fibrinogen to fibrin		
	Abscess formation		
Von Willebrand factor binding protein	Activation of prothrombin	Rabbit skin	[90,113]
	Abscess formation		

**Table 3:** Key cell wall-anchored protein virulence factors involved in *S. aureus* skin-and-soft-tissue infection.

Virulence Factor	Function	Models	References
Cell wall-anchored proteins			
Sortases (SrtA, SrtB)	Covalent attachment of surface proteins to cell wall	Mouse skin	[114,115]
	Abscess formation		
Clumping factors (ClfA, ClfB, SdrC, SdrD, SdrE)	Binding fibrinogen (ClfA, ClfB, SdrE), desquamated epithelial cells (SdrC, SdrD)	Mouse skin, intravenous Rabbit skin	[90,105,114,115,116]
	Early dissemination (ClfA, ClfB)		
	Abscess formation (SdrD)		
Near iron transporters (IsdA, IsdB)	Binding and transporting heme Abscess formation	Mouse skin	[90,105,116,117,118,119]
		Rabbit skin	
		Monkey skin	
Fibronectin-binding proteins A and B (FnBPA, FnBPB)	Adhesion to extracellular matrix	Human corneocytes, human immortal keratinocytes, skin explants, mouse mast cells	[90,114,116,120,121,122,123,124,125]
	Internalization of bacteria		
	Abscess formation		
Extracellular adherence protein (Eap)	Adherence to extracellular matrix	Human HaCaT keratinocytes	[90,116,124,126,127]
	Internalization by eukaryotic cells		
	Biofilm formation		
	Abscess formation		
Extracellular matrix protein (Emp)	Binding to extracellular matrix	Human skin	[90,116,127,128]
	Biofilm formation		
	Abscess formation		
Peptidoglycan-anchored proteins (SasX)	Covalent attachment to peptidoglycan	Mouse skin	[129,130,131]
	Colonization		
Protein A	Binding IgG, TNF receptor 1, von Willebrand factor	Mouse skin Human HaCaT keratinocytes	[90,92,114,132,133,134,135,136,137,138,139,140]
	Evasion of phagocytosis		
	Superantigenic factor for B cell apoptosis		
	Pro-inflammatory cytokine expression		
	Atopic dermatitis		

## Data Availability

No new data were created or analyzed in this study. Data sharing is not applicable to this article.
